# Time to Loss of Behavioral and Brainstem Responses of Ducks following Non-Stunned Slaughter

**DOI:** 10.3390/ani11123531

**Published:** 2021-12-11

**Authors:** Alexandra Friedman, Filipe Antonio Dalla Costa, Osmar Antonio Dalla Costa, Alicia Godsell-Ryan, Troy John Gibson

**Affiliations:** 1 Department of Pathobiology and Population Sciences, Royal Veterinary College, University of London, Hawkshead Lane, Hatfield AL9 7TA, UK; afriedman7@rvc.ac.uk (A.F.); agodsell6@rvc.ac.uk (A.G.-R.); 2 Department of Strategy and Innovation, MSD Animal Health, São Paulo 01000-000, Brazil; filipe.dallacosta@merck.com; 3 Embrapa Suínos e Aves, Concórdia 89700-991, Brazil; osmar.dallacosta@embrapa.br

**Keywords:** animal welfare, non-stunned slaughter, halal, duck, slaughterhouse technique, consciousness

## Abstract

**Simple Summary:**

For routine poultry slaughter, animals are stunned prior to the act of slaughter to prevent pain and distress. Halal slaughter requires either reversible or no stunning before performance of the neck cut. This study measured how long it takes for ducks to lose consciousness following non-stunned slaughter in a commercial processing plant. The study found that ducks take up to 383 s to lose consciousness. The performance of a cut higher on the neck resulted in a faster time to loss of consciousness. This study is the first investigation of the time to loss of consciousness following non-stunned slaughter of ducks in commercial conditions. These results could be used to improve the welfare of ducks during non-stunned slaughter, such as recommending a higher neck cut and ensuring appropriate waiting periods between slaughter and birds entering the scalding tanks.

**Abstract:**

Non-stunned slaughter has been extensively described for other farmed species but there has been limited research on waterfowl. The study assessed 34 White Pekin ducks (*Anas platyrhynchos*) (study 1) in a non-stunned halal slaughterhouse in Brazil for time to loss of consciousness using various behavioral and brainstem indices (balance, cranial nerve reflexes, and muscle tension) and assessed the relationship between extent of clotting, location of neck cut, level of damage to neck vessels/tissues, and the time to onset of unconsciousness. In addition, operator practices were separately observed and neck pathology following the cut was examined in 217 carcasses after bleeding (study 2). In study 1 following the neck cut there was a wide variation between birds in the time to loss of behavioral and brainstem indices, ranging from 20 to 334 and 20 to 383 s for neck and beak tension, respectively. The median time to loss of balance following the neck cut was 166 ± 14 (22–355) seconds. There was a moderate correlation (R = 0.60 and 0.62) between distance of the neck cut and time to loss of balance and neck tension, respectively. This is the first investigation of the time to loss of consciousness following non-stunned slaughter of ducks in commercial conditions. The findings could be used to improve the welfare of ducks during non-stunned slaughter, such as recommending performance of the neck cut closer to the jaw line and ensuring appropriate waiting periods between slaughter and birds entering the scalding tanks.

## 1. Introduction

Poultry and waterfowl are generally stunned prior to slaughter with exposure to controlled atmosphere or electrical stunning. In some countries there is a derogation that allows the performance of non-stunned slaughter for religious slaughter [1]. During non-stunned slaughter, a ventral neck-incision is made, which transects skin, muscle, trachea, oesophagus, sensory nerves, connective tissue, carotid arteries, and jugular veins. There are a number of potential welfare concerns following non-stunned slaughter, which include distress associated with restraint [2,3,4], pain due to the neck cut [5,6,7,8], aspiration of blood into the upper respiratory tract, and distress due to delays in the time to onset of unconsciousness [2,9,10]. Most studies on non-stunned slaughter of birds have focused on chickens, with only limited research on ducks.

Time to loss of consciousness may be affected by a number of factors such as the following: 1. Extent of vessel severance; the presence of intact carotids has been associated with a delayed time to unconsciousness in a number of species [11,12,13], with previous estimates in non-stunned poultry suggesting 10% of birds may be affected [12]; 2. Extent of clotting; clotting prevents efficient bleeding, which is the cause of death during non-stunned slaughter [14] and hindrance of this may delay the time to unconsciousness [15]; and 3. Sharpness of the knife; the use of blunt knives has been shown to result in an increase of electroencephalographic (EEG) indices in cattle associated with pain [16], increases the number of cuts required to slaughter cattle [14], and increase endothelial cell damage, which activates clotting factors [17]. There is evidence that knife sharpness is more dependent on operator sharpening skill than knife material [18]. 

Halal slaughter of poultry requires full section of both carotid arteries and jugular veins, as well as the oesophagus and trachea [19]. A “razor-sharp” knife that is twice as long as the neck width of the bird (poultry, including waterfowl) is recommended in order to complete the neck cut in a single stroke and therefore ensure swift slaughter [2,14,20]. There is currently no evidence-based guidance on the ideal location of the neck cut for chickens or ducks. The Humane Slaughter Association (HSA) recommends slaughtering birds with the knife positioned just below the jawbone [21]. 

Assessing the time to unconsciousness and/or death in poultry may involve a variety of proxy measures. Previous studies on non-stunned poultry slaughter included loss of heartbeat [20] to indicate death, but cardiac arrest is often preceded by loss of consciousness. From an animal welfare perspective, the loss of consciousness or related proxies should be the central focus of assessment and not loss of cardiac function. Indirect measures of brain function that could support assessment of consciousness include the presence of nictitating membrane, palpebral and corneal reflexes [9,22,23,24]; recovery and maintenance of muscle tone (neck and beak muscle tension) [22,24,25]; signs of vestibular and cerebellar motor control (posture and balance) [9,25,26]; rhythmic breathing [23,24,25]; and evoked responses to noxious stimuli (comb and skin pinching) [20,27]. In broilers, loss of neck tension and inability to maintain a sitting position as a proxy for loss of consciousness has been supported by EEG data [26]. Assessing a combination of cranial nerve reflexes, medullary functions, and post-death behaviors is crucial [28,29], as no single behavior or reflex can be relied upon to determine consciousness or unconsciousness. Instead, the combination of these reflexes allows a more robust assessment of brain and brainstem function to determine whether an individual bird is conscious or unconscious, or transitioning between these states. 

There is evidence that ducks have different physiological responses to slaughter [30] and require higher electrical current at stunning [27,31,32]. Ducks have been reported as having an unusually extensive connective tissue sheath surrounding the jugular vein [33] and deeply embedded carotid arteries [21]. Finally, White Pekin ducks have been shown to have a small alternative circulatory supply to the brain, including via the vertebral arteries [34], which were estimated to contribute approximately 2% of brain circulation. Therefore, results from chicken based research cannot be directly transferred to duck slaughter. With both stunned and non-stunned methods, it is critical that birds are clinically dead (loss of behavioral, brainstem and spinal reflexes, loss of brain activity, and in cardiac arrest) before entering the scalder [19,20], to prevent unacceptable welfare compromise. Given these factors and the increase in halal meat demand in Europe and elsewhere over the past 20 years [35] and projections for demand to increase by another 5% in the next five years [36], there is a need to investigate non-stunned slaughter in ducks and how it can be managed efficiently to reduce the potential welfare impact. 

The aim of this study was to assess time to loss of behavioral and brainstem reflexes of ducks during non-stunned halal slaughter, compared with cut position and pathology. 

## 2. Materials and Methods

### 2.1. Study 1: Time to Loss of Behavioral and Brainstem Responses

Thirty-four White Pekin ducks (*Anas platyrhynchos*) were assessed by a single experienced observer in an 1800 ducks/h commercial non-stun halal slaughterhouse in Brazil over a period of two days in December 2019. As per routine practice, conscious birds were hung and shackled by their feet and were further restrained in a cone. A single, diagonal cut was made across the neck from the bird’s right-hand side by an operator, with the aim of severing the carotid arteries, jugular veins, trachea, oesophagus, and connective tissues of the neck ventral to the vertebral column. At random intervals the line was then stopped, and individual birds were removed and placed on a table on their feet. It took an average of 15 s from the neck cut to removing the bird from the line. They were then tested in the same sequence for nictitating reflexes, beak and neck tension, evidence of breathing, ability to stand (time to collapse), and balance (complete loss of coordinated balance) (Table 1). Assessments were repeated in the same sequence until all reflexes were absent. Extent of severance of the main tissues (trachea, oesophagus, and spinal cord) and vessels (jugular veins and carotid arteries) of the neck (assessed as intact, nicked, partially severed, and completely severed) was recorded, as were location of neck cut (distance from right-hand side of the jawbone), extent of clotting, and presence of convulsions. Vessels and tissues defined as nicked, had a small cut with their structure remaining intact. Partially severed vessels and tissues had greater than 50% of the structure severed, preventing the transportation of blood. Time of onset and duration of convulsions could not be recorded due to issues differentiating wing flapping from convulsions when birds were removed from the shackle line. All assessments were performed by the same experienced observer.

The assessments were recorded on an iPhone X (Apple, Cupertino, CA, USA) for detailed analysis. Video analysis and time elapsed to loss or recurrence of each reflex was recorded from the point of removing the bird from the line to maintain consistency. 

### 2.2. Study 2: Operator Behavior and Post Slaughter Cut Pathology

The two slaughtermen on the line were also studied. Frequency of knife sharpening, number of strokes on the sharpener, and number of washes of the knife in between birds were recorded. The operator data were not recorded in correlation to the reflex analysis of the birds in study 1. In addition, after the bleeding area and immediately prior to the scalding tank the carcasses of 217 birds were assessed for vessel/tissue severance and neck cut position, using the same criteria as described for study 1. These birds were linked to the individual operators (56 and 116 for operators A and B, respectively). The number of birds per operator was determined by the break patterns in the slaughterhouse.

### 2.3. Statistics

All statistical analyses were conducted using RStudio for Mac, version 1.3.959 (RStudio, Boston, MA, USA). Association between operator behavior, cut position and tissues/vessels cut was analyzed using a Wilcoxon Two-sample Test for non-parametric data. The relationship between cut distance and each of the reflexes measured was assessed via a Pearson’s correlation. A Welch’s Two-sample T-Test was conducted to assess the difference between reflexes and presence of clotting. Association between extent of clotting and cut distance was evaluated using a two-way analysis of variance (ANOVA) assuming non-variant means.

## 3. Results

### 3.1. Study 1: Time to Loss of Behavioral and Brainstem Responses

On average the first reflex to cease was ability to stand (collapse), and the last was nictitating membrane reflex (Table 2). Sixteen (47%) birds had at least one behavioral or brainstem reflex after 180 s. All reflexes were lost by 383 s. Four birds (12%) had one or more vessels at least partially intact (3 carotid arteries, 1 jugular vein) (Table 3). Spinal cord damage could not be assessed due to insufficient lighting. Nictitation was the most variable reflex assessed: 61% of birds lost and gained the ability to nictitate at least once during assessment. Rhythmic breathing was not observed in any of the birds. Convulsion-like movements were observed in 24/35 birds (68%) by the time the recording ended (once all reflexes were absent). 

**Figure 1:**
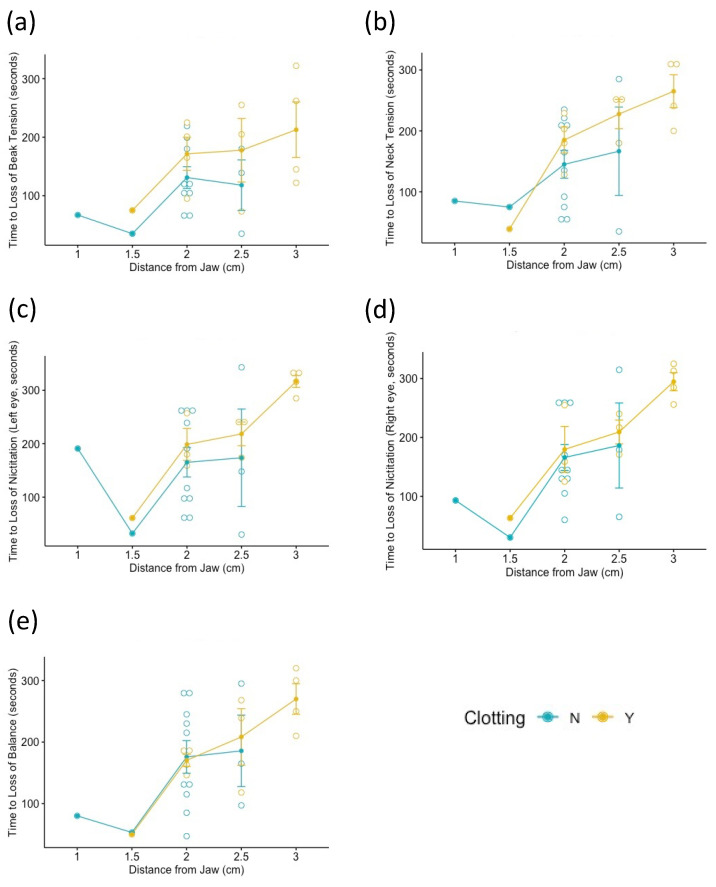
Relationship between distance from right jawbone to neck cut (X axis) and the time to loss of a reflex (Y axis), grouped by presence of clotting (yellow = clotting present, blue = clotting not present). Each point represents a data point (bird). The reflexes are as follows: (**a**) Total loss of beak tension, (**b**) total loss of neck tension, loss of (**c**) left and (**d**) right nictitating reflex, and (**e**) loss of balance. None of the results were significant (*p =* 0.208, 0.303, 0.398, 0.719, and 0.880, respectively).

The average cut position was 2.2 ± 0.5 (SD) (range 1 to 3) cm from the base of the right aspect of the jaw. The four reflexes tested (beak tension, neck tension, loss of balance, and nictitation) were significantly influenced by the neck cut location (Table 4). When clotting was present there was no significant difference in time to loss of both neck tension and balance (*p* = 0.208 and 0.88, respectively) (Figure 1, Table 4). However, there was a significant difference when comparing presence and absence of clotting alone for three out of the four reflexes: beak tension (*p =* 0.047), neck tension (*p =* 0.010), and nictitation (left *p =* 0.022 and right *p =* 0.035 eye).

**Figure 2:**
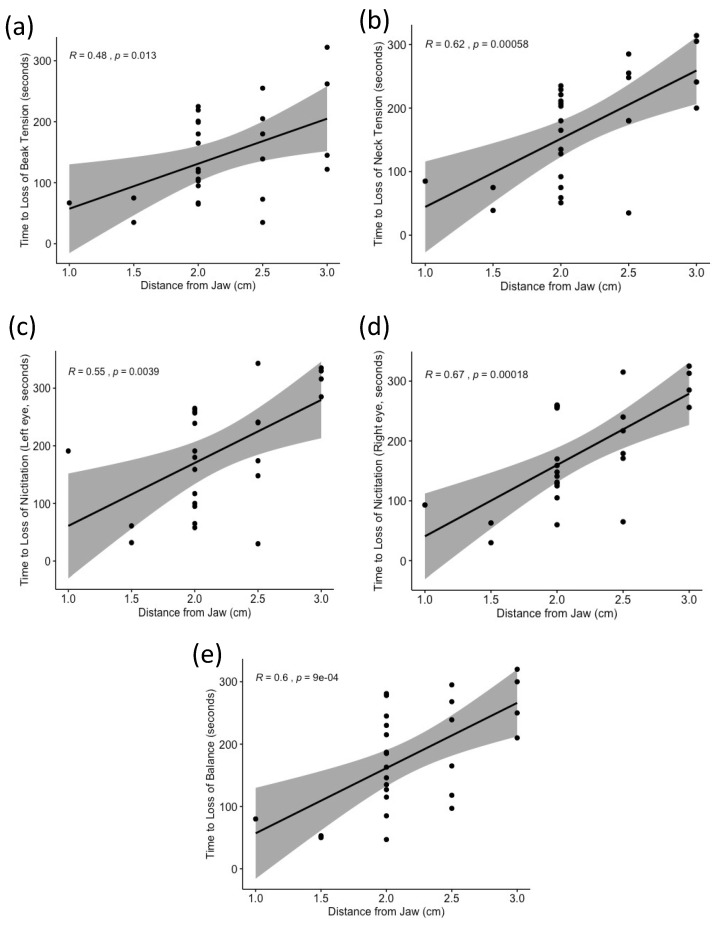
Correlation with confidence intervals between cut distance from jawbone (in centimeters) and behaviors/brainstem reflexes: (**a**) Time to loss of beak tension, (**b**) time to loss of neck tension, time to loss of nictitation in the (**c**) left and right (**d**) eye, and (**e**) time to loss of balance and. Each point represents a data point (bird). All correlations were significant at the *p* < 0.05 level.

There were significant positive moderate correlations between the distance of the neck cut from the jawbone and the time to loss of nictitating reflex (left eye R = 0.55, *p =* 0.004; right eye R = 0.67, *p* < 0.001), beak tension (R = 0.48, *p =* 0.013), balance (R = 0.60, *p =* 0.001), and neck tension (R = 0.62, *p =* 0.006) (Figure 2). There was no significant correlation between distance from the jaw and time to collapse (R= 0.26, *p =* 0.200).

### 3.2. Study 2: Operator Behavior and Post Slaughter Cut Pathology

The number of birds slaughtered between knife sharpens was significantly different between operators A and B (median 32 birds vs. 52 birds, respectively, *p =* 0.011). Operator A and B also were significantly different in the number of strokes on the knife sharpener (median 5 and 3 strokes, respectively, *p =* 0.036). Operator A therefore sharpened the knife more often and with more strokes on the sharpener than Operator B. There was no significant difference between knife washes under the tap (*p =* 0.360). 

The mean cut position for both operators was 2.1 ± 1.6 (SD) cm from the base of the right aspect of the jaw. There was no significant difference in cut position between the two operators (A 1.9 ± 0.4 and B 2.0 ± 0.5 cm; *p =* 0.369). The trachea and oesophagus were completely severed in all birds (Table 5). Only one bird had unilateral severance of the carotid arteries (left was intact), while three birds had nicked (1 left carotid) or partially severed (1 left and 1 right) carotids unilaterally, with the other carotid completely severed. Eighty three percent of birds had a partially severed spinal cord. There was no significant difference between operators in cut pathology.

## 4. Discussion

To the authors’ knowledge this is the first published study of time to loss of behavioral and brainstem reflexes following non-stunned slaughter of ducks in commercial conditions. Much of the previous limited research on slaughter of non-stunned birds has focused on chickens and turkeys, principally broilers. 

In the study of the time to loss of behavioral and brainstem reflexes (study 1), the first mean behavior that was absent following the neck cut was the ability to maintain posture, this was followed by muscle tone (beak and neck tension), balance and then nictitating membrane reflex. The loss of the nictitating membrane reflex has been previously suggested to be an indicator of brainstem death as opposed to consciousness in chickens, turkeys and rabbits [29,37,38]. Nictitation can be evoked in anaesthetized animals [39,40] and is generally the last reflex to be lost following non-stunned slaughter [39]. Because of this it should not be relied upon as a sole indicator of consciousness/unconsciousness; instead it should be used to indicate brainstem function, which could support consciousness. 

Time to loss of coordinated muscle tension and posture has been previously used as an indicator of onset of unconsciousness following non-stunned slaughter of broilers [9]. In broilers the mean time to loss of posture has been reported to be 14 s (*n* = 41 birds), with some birds taking up to 26 s [9]. This contrasts with the mean time of 144 (max 383) and 166 (max 355) seconds it took ducks in the current study to lose muscle tone (beak tension and balance, respectively). This difference may relate to differences in physiology and anatomy between the species. Diving water birds have been previously reported as having adaptions that allow them to remain submerged when active for extended periods [41]. Bryan and Jones [42] reported that when subjected to apnoeic asphyxia, ducks were able to maintain spontaneous EEG activity fivefold longer than chickens, with the EEG becoming isoelectric at a mean of 338 and 63 s for ducks and chickens respectively. These times to loss of EEG activity are comparable to the behavioral/reflex data of ducks in the current study with the longest time to unconsciousness. However, care must be taken with concluding mechanisms, as several authors have demonstrated that ducks do not have any unique biochemical tolerance to cerebral hypoxia, rather the difference is likely mediated through the oxygen-conserving cardiovascular reflex, anaerobic metabolism and changes in metabolic rates [13,41,42]. These physiological mechanisms would have limited influence on exsanguination induced cerebral hypoxaemia experienced during slaughter, other than supporting continued oxygenation via the vertebral arteries. However, this is unlikely as Fedde and Guffy [34] estimated for ducks that the vertebral arteries only contribute 2% to cerebral circulation. Furthermore, Gregory and Wotton [13] did not observe distinct differences in spontaneous EEG or visual evoked potentials of chickens and ducks following bilateral exsanguination of the carotid arteries. However, care must be taken in making comparisons with this work as these studies were conducted on barbiturate anaesthetized and ventilated birds [13]. Anaesthesia would have suppressed normal baseline EEG activity and influenced the electrophysiological responses to cerebral hypoxia.

In study 1, there was extensive clotting in the cut region in many birds and this was associated with delays in onset of loss of assessed reflexes. Similar clotting was also seen in study 2 but not recorded. Duck blood has been previously reported to have higher specific activity of fibrin stabilizing factor (factor XIII) compared with chicken blood [43]. This transglutaminase is involved in clotting and helps to increase clot breaking strength. Potentially, this clotting activity could have impeded blood loss and contributed to the delay in time to unconsciousness. Compared with chickens, ducks have an extensive connective tissue sheath surrounding the jugular veins [33] and deeply embedded carotid arteries [21]. These factors complicate cutting performance and potentially the connective tissues provide a surface for enhanced clotting. Further work is required to clarify and/or identify the physiological/anatomical mechanisms associated with the differences in time to loss of consciousness between the species.

Rhythmic breathing was not observed in any of the ducks in study 1. In this context it may be interpreted as a very early loss of consciousness, but that is not supported by the presence of other behavioral and reflex indices. It is more likely that the lack of observed rhythmic breathing is a result of trachea severance and the inability to fill the air sacs. The birds would also often collapse and subsequently have intermittent periods of regaining the ability to stand. This phasic return of posture has also been observed in cattle following non-stunned slaughter [11,44]. Collapse is an early indicator of loss of consciousness, and it has been hypothesized that the phasic return of the ability to maintain posture may be due to the animal’s restoration of neurological function [11,45]. In study 1, neck cut position influenced the time to loss of indices, with each additional centimeter further away from the jawbone adding approximately 1 min to the time to loss of behavioral/brainstem reflexes. In addition, all four ducks with a 3 cm cutting distance had extensive clotting, suggesting that the cut-to-jaw distance may influence clotting. However, due to the small sample size it was not possible to test this hypothesis further. The performance of a high neck cut has been previously shown to reduce time to loss of consciousness in cattle during non-stunned slaughter [44,46]. In chickens and ducks the carotid arteries run parallel to the jugulars within the thoracic inlet and are located deep within the neck musculature [21], before becoming more exteriorized closer to the jaw following divergence of the left and right arteries [47]. Based on the current findings, it is hypothesized that performance of a higher neck cut in ducks could potentially reduce the time to loss of consciousness. 

A potential limitation of study 1 was the small sample size, which was unavoidable due to the commercial setting in which this study was conducted. Potentially, the small sample size could have led to a misrepresentation of the mean time to unconsciousness. However, from an animal welfare perspective, the focus is on the experience of the individual animal, and hence the delays reported in the study in the time to loss of consciousness in some ducks following non-stunned slaughter are important from a precautionary principle approach. This is especially the case given the lack of data in this area. It is unlikely that a larger sample size would have influenced this aspect of the results. In the ducks that were examined for behavioral and brainstem reflexes, it was not possible to assess damage to the spinal cord due to insufficient lighting. However, in study 2, which assessed carcasses prior to the scalding tank, 83% of birds had partially severed spinal cords. Based on these results it was probable that a proportion of birds in study 1, which examined behavioral/brainstem reflexes post neck cutting, had some level of damage to their spinal cords. This would have potentially affected the expression of certain behavioral/reflexes, particularly breathing and those involving coordination of muscle and vestibular activity. This may have influenced the results of some birds, potentially skewing them to suggest an earlier time to loss of consciousness (based on the integrated behavioral/brainstem indices). The recording of EEG would have allowed the comparison of brain activity with behavioral/brainstem reflexes, potentially identifying birds with damaged spinal cords. However, this was not possible due to this study being conducted in a commercial slaughterhouse environment. 

European legislation requires poultry to remain unconscious for the entire duration of the slaughter and post-slaughter process [48]. Given the variation in both efficacy of duck stunning methods [31,32] and time to unconsciousness (up to 383 s), there is the potential that some birds can recover from stunning prior to cerebral hypoxia induced brain death. Furthermore, to meet halal laws, Shahdan et al. [20] recommend that in non-stunned poultry slaughter, a period of 9.5 min from slaughter to further processing is provided to ensure death occurs prior to birds entering the scalding tank. Their field survey found poultry to take between 3 and 9 min before entering the scalder. In study 1, it was found that 16/34 ducks (47%) had at least one sign of consciousness after 180 s. In this slaughterhouse the time from neck cutting to the scalding tank was approximately 5 min. Hence, there is the potential, based on the data from this study that some birds may still have retained some level of consciousness up to the point of entering the scalding tank. This would result in significant pain and distress prior to the loss of consciousness. The results of study 1 further support the recommendation by Shahdan et al. [20] that post neck cutting waiting periods of at least 383 s (6.5 min) prior to birds entering the scalding tank are required during commercial non-stunned slaughter of ducks.

The disparities in the operator behavior in study 2, including differences in knife sharpening frequency and extent of sharpening, reflect differences that can have an impact on neck cut performance, which can subsequently cause further pain and delays in the time to loss of consciousness. Imlan et al. [16] reported in cattle slaughtered with blunt knifes, that EEG indices associated with pain were significantly increased compared with animals slaughtered with a sharp knife. In addition, any imperfections to the knife such as nicks could cause tearing of tissues resulting in further pain and distress. Further studies with larger sample sizes are needed to better understand how knife sharpening behaviors, variations in operators and training can influence time to loss of consciousness and would add clarity to this data. In study 2, although the majority of birds had both carotids severed, there was one bird with unilateral carotids severance. Unilateral carotid and jugular severance has been shown to double the time to loss of spontaneous EEG activity (122 s) and visual evoked potentials (302 s) in anaesthetized chickens [13]. For this bird the severance of only one carotid artery could have led to prolonged pain and distress during the bleeding process, and the potential for entering the scalding tank while still conscious. 

## 5. Conclusions

Ducks take markedly longer to lose consciousness following non-stunned slaughter than previously considered. Delays in the time to unconsciousness are likely to result in the ducks experiencing pain and distress, and may result in still conscious birds entering the scalder. Performance of a neck cut closer to the jaw may reduce the time to unconsciousness and death in ducks, potentially reducing the welfare compromise associated with the delayed time to loss of consciousness during slaughter without stunning. 

## Figures and Tables

**Table 1:** Ethogram defining each behavior recorded after the neck cut, assessments were repeated in the same sequence until all reflexes were absent.

Behavior	Definition
Loss of Nictitating Membrane Reflex	Exposed corneal and corner of eye are stimulated with a probe to elicit a nictitating membrane reflex. Nictitating membrane fails to close upon stimulation.
Loss of Beak Tension	No resistance when manually opening beak.
Loss of Neck Tension	Examined by raising the head and neck, followed by the withdrawal of support with assessment of maintenance of muscle tone and/or controlled movement of the head. Unable to keep neck in upright position.
Loss of Rhythmic Breathing	Observation and palpation of the posterior aspect of the abdominal cavity for signs of rhythmic air sac filling.
Collapse	Bird collapses into a sitting posture after being placed in a standing position.
Loss of Balance	Bird is placed on its feet in a standing or sitting posture (if not weight bearing) and manually tilted off balance (laterally) for observation of righting of posture. Bird is recumbent with no evidence of postural control or righting reflex.
Convulsion-like movements *	Involuntary contraction of skeletal muscle (includes paddling motions).

* Convulsions-like movements were measured as either present or absent due to similar behaviors having a possible confounding effect, such as struggling to stand/escape and various aetiologies of convulsions (anoxia to brain vs. muscle).

**Table 2:** Time to loss of behaviors and brainstem reflexes (*n* = 34) following the neck cut, all values include the 15 s it took from the neck cut to the beginning of assessment.

	Time to Loss (s)	
Reflex	Mean ± SE	Range (Min–Max)
Collapse	73 ± 10	0–210
Beak Tension	144 ± 14	20–383
Neck Tension	159 ± 14	20–334
Balance	166 ± 14	22–355
Nictitation (Right Eye)	170 ± 15	15–365
Nictitation (Left Eye)	176 ± 17	15–366
Rhythmic breathing	-	-

**Table 3:** Neck pathology of the ducks assessed for loss of behavioral/brainstem responses (*n* = 34).

	Intact	Nicked	Partially Severed	Completely Severed
Trachea	0 (0%)	0 (0%)	0 (0%)	34 (100%)
Oesophagus	0 (0%)	0 (0%)	1 (3%)	33 (97%)
Right Carotid artery	2 (6%)	0 (0%)	1 (3%)	31 (91%)
Left Carotid artery	0 (0%)	0 (0%)	0 (0%)	34 (100%)
Right Jugular vein	0 (0%)	0 (0%)	1 (3%)	33 (97%)
Left Jugular vein	0 (0%)	0 (0%)	0 (0%)	34 (100%)

**Table 4:** Relationship between behavioral and brainstem reflexes and neck cut distance and clotting (*n* = 34), values marked with a * are significantly different at *p* < 0.05 level.

Reflex	Parameter	Df	Sum of Squares	Mean Square	F Value	*p* Value
Beak Tension	Cut Distance	1	32,879	32,879	7.16	0.014 *
	Clotting	1	7734	7734	1.68	0.208
	Cut Distance: Clotting	1	368	368	0.08	0.780
Neck Tension	Cut Distance	1	69,887	69,887	15.38	<0.001 *
	Clotting	1	5041	5041	1.11	0.303
	Cut Distance: Clotting	1	2942	2942	0.65	0.429
Nictitation (Left Eye)	Cut Distance	1	172,129	72,129	10.61	0.004 *
	Clotting	1	5047	5047	0.74	0.398
	Cut Distance: Clotting	1	15,710	15,710	2.31	0.143
	Cut Distance	1	85,694	85,694	18.51	<0.001 *
Nictitation (Right Eye)	Clotting	1	616	616	0.13	0.719
	Cut Distance: Clotting	1	2729	2729	0.60	0.451
Balance	Cut Distance	1	66,396	66,396	13.24	0.001 *
	Clotting	1	117	117	0.023	0.880
	Cut Distance: Clotting	1	1442	1442	0.288	0.597

**Table 5:** Post slaughter cut pathology prior to the scalding tank (*n* = 217).

	Intact	Nicked	Partially Severed	Completely Severed
Trachea	0 (0%)	0 (0%)	0 (0%)	217 (100%)
Oesophagus	0 (0%)	0 (0%)	0 (0%)	217 (100%)
Right Carotid artery	0 (0%)	0 (0%)	1 (1%)	216 (100%)
Left Carotid artery	1 (1%)	1 (1%)	1(1%)	214 (99%)
Right Jugular vein	0 (0%)	2 (1%)	1 (1%)	214 (99%)
Left Jugular vein	0 (0%)	0 (0%)	4 (2%)	213 (98%)
Spinal cord	38 (18%)	0 (0%)	179 (83%)	0 (0%)

## Data Availability

The data presented in this study are available on request from the corresponding author.
